# GAGA Factor Maintains Nucleosome-Free Regions and Has a Role in RNA Polymerase II Recruitment to Promoters

**DOI:** 10.1371/journal.pgen.1005108

**Published:** 2015-03-27

**Authors:** Nicholas J. Fuda, Michael J. Guertin, Sumeet Sharma, Charles G. Danko, André L. Martins, Adam Siepel, John T. Lis

**Affiliations:** 1 Department of Molecular Biology and Genetics, Cornell University, Ithaca, New York, United States of America; 2 Department of Biological Statistics and Computational Biology, Cornell University, Ithaca, New York, United States of America; University of Chicago, United States of America

## Abstract

Previous studies have shown that GAGA Factor (GAF) is enriched on promoters with paused RNA Polymerase II (Pol II), but its genome-wide function and mechanism of action remain largely uncharacterized. We assayed the levels of transcriptionally-engaged polymerase using global run-on sequencing (GRO-seq) in control and GAF-RNAi Drosophila S2 cells and found promoter-proximal polymerase was significantly reduced on a large subset of paused promoters where GAF occupancy was reduced by knock down. These promoters show a dramatic increase in nucleosome occupancy upon GAF depletion. These results, in conjunction with previous studies showing that GAF directly interacts with nucleosome remodelers, strongly support a model where GAF directs nucleosome displacement at the promoter and thereby allows the entry Pol II to the promoter and pause sites. This action of GAF on nucleosomes is at least partially independent of paused Pol II because intergenic GAF binding sites with little or no Pol II also show GAF-dependent nucleosome displacement. In addition, the insulator factor BEAF, the BEAF-interacting protein Chriz, and the transcription factor M1BP are strikingly enriched on those GAF-associated genes where pausing is unaffected by knock down, suggesting insulators or the alternative promoter-associated factor M1BP protect a subset of GAF-bound paused genes from GAF knock-down effects. Thus, GAF binding at promoters can lead to the local displacement of nucleosomes, but this activity can be restricted or compensated for when insulator protein or M1BP complexes also reside at GAF bound promoters.

## Author Summary

Transcriptional regulation is critical for proper gene expression in response to environmental changes and developmental programs. Eukaryotes have evolved multiple mechanisms by which transcription factors regulate transcription. One mechanism is the reorganization of chromatin to allow Pol II recruitment. Another is the release of promoter-proximal paused Pol II, where Pol II transcription that is halted 20–60 bases downstream of the transcription start site (TSS) is allowed to enter into productive elongation through the gene body. The Drosophila transcription factor GAF binds to genes that undergo pausing and interacts with nucleosome remodelers and the pausing factor NELF. Thus, GAF can regulate multiple points necessary for transcription, but its mechanistic role is not fully understood genome-wide. We depleted GAF from cells and examined the genome-wide changes in Pol II and nucleosome distributions across genes. We found that GAF depletion reduces polymerase density at genes where GAF binds just upstream of the TSS, and results in nucleosomes moving into the promoter region. Our results show that GAF is important for maintaining the promoter accessibility, allowing Pol II to be recruited to promoters and enter the pause sites downstream of the TSS. Thus, GAF is critical for providing the chromatin environment necessary for the proper control of gene expression.

## Introduction

Transcription is controlled by transcription factors (TFs) that modulate various steps in the transcription process. Two major points of transcription regulation are recruitment of Pol II to a preinitiation complex (PIC) and promoter-proximal pausing. PICs form when general transcription factors bind to accessible nucleosome-free promoters and recruit Pol II. TFs can change the rate of PIC formation by altering either nucleosome placement on promoters or Pol II recruitment [1]. In addition, many genes are regulated after Pol II recruitment by the controlled release of a stable paused Pol II, which is typically located in the promoter-proximal region 20–60bp downstream of the transcription start site [2]. TFs can stimulate release Pol II from the pause by recruiting, directly or indirectly, P-TEFb kinase that modifies the paused Pol II complex, allowing it to efficiently transcribe across the gene [3].

GAF, encoded by the gene *Trithorax-like* (*Trl*), is a Drosophila sequence-specific TF that is associated with the promoters of many genes [4]. GAF was first identified as a regulator of developmental genes and binds GA repeats [5–9]. The GAF DNA binding domain is composed of a basic-rich region followed by a C2–H2 zinc finger that binds DNA sequences as short as GAG or the longer sequence of GAGAG in vitro [5,6,10]. However, in vivo bound regions generally have clusters of GAGA elements [11,12]. In addition to the DNA-binding domain, GAF has a BTB/POZ domain that mediates interactions with other proteins, and allows GAF to homodimerize or heterodimerize with other BTB/POZ-containing factors [13–17]. GAF also has a polyQ domain. Its function is not well-understood, but has been reported to act both as a transcription activator [18,19] and as a multimerization domain that can influence DNA binding [20,21].

Genome-wide studies have identified many genes bound by GAF [4,11,22–24], and GAF binding is enriched on paused genes [4,25,26]. In addition, transgenic reporter genes have transcriptionally-engaged polymerase in their promoter-proximal regions under basal conditions when GAGA elements are present [27,28]. These results suggest that GAF plays a role in establishing paused polymerase.

Several reports support a role of GAF as an anti-repressor for genes [29]. The GAF anti-repressor function is proposed to maintain promoters in an accessible state [30]. GAF can interact with several nucleosome remodelers, including NURF, ISWI, and BPAP, and displace adjacent nucleosomes to make DNA accessible regions [30–33], but this function of GAF has not been investigated in a genome-wide manner.

Here, we examine the role of GAF in transcriptional regulation and nucleosome positioning genome-wide, using global run-on sequencing (GRO-seq) to map transcriptionally-engaged polymerases and MNase-seq to map nucleosome positions in control and GAF-RNAi depleted Drosophila S2 cells. Also, we define GAF binding sites at high resolution and assess their sensitivity to GAF knock-down using ChIP-seq. This allows GAF binding in promoters to be correlated with its effects on transcription and pausing and other factors that function redundantly to GAF or protect genes from the effects of GAF knock-down. Finally, MNase-seq mapping of nucleosomes genome-wide in control and GAF-RNAi cells supports a mechanism by which bound GAF maintains a nearby nucleosome free region at both the promoters of many genes and non-promoter sites.

## Results

### GAF is important for promoter-proximal pausing on *Hsp70*

**Fig 1:**
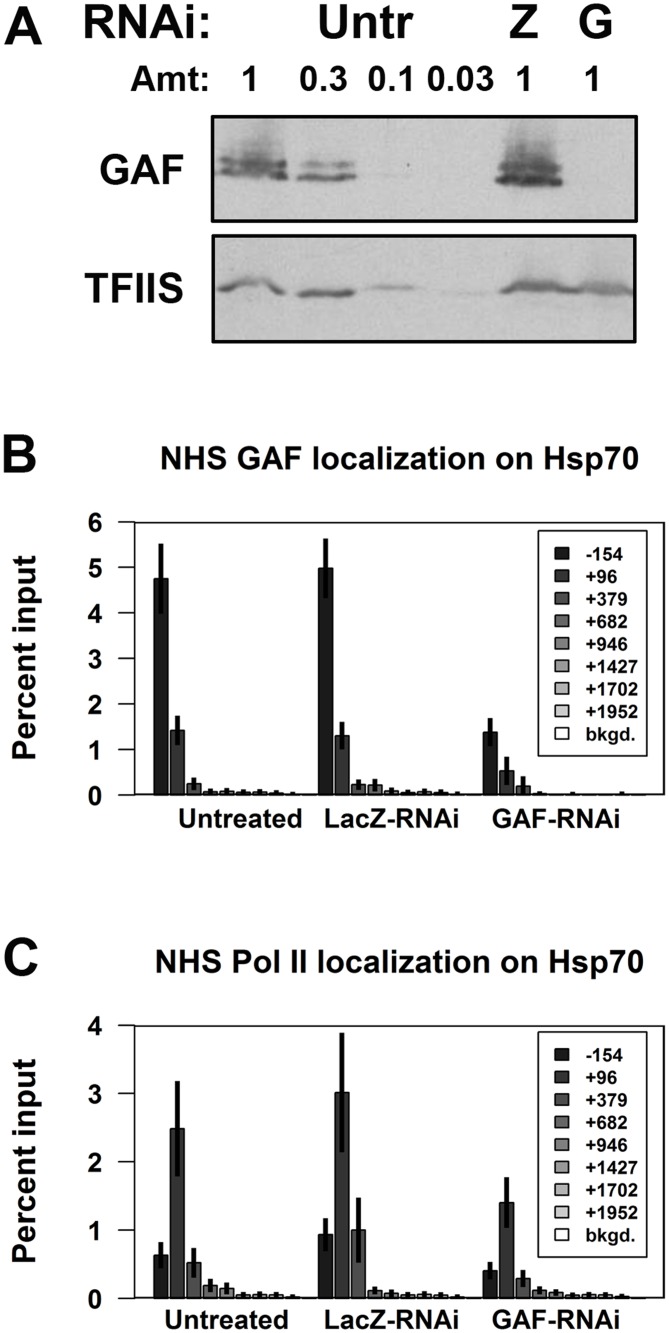
Depletion of GAF reduces paused polymerase on NHS *Hsp70*. **(A)** Western blot of whole cell extracts from Untreated (Untr), LacZ-RNAi (Z), and GAF-RNAi (G) cells for GAF and a loading control, TFIIS (1 is equivalent to 1x10^6^ cells). **(B)** ChIP-qPCR for GAF on *Hsp70* in non-heat shock (NHS) Untreated, LacZ-RNAi, and GAF-RNAi cells. **(C)** ChIP-qPCR for Pol II subunit Rpb3 on *Hsp70* in NHS Untreated, LacZ-RNAi, and GAF-RNAi cells. The legends indicate the center of each primer set relative to the TSS. The error bars represent the standard error from at least 3 experiments.

We initially examined the role of GAF in pausing on the prototypical paused genes, *Hsp70*. Under basal (non-heat shock, NHS) conditions, GAF is bound to the *Hsp70* promoters and Pol II transcribes 20–40 bases downstream from the transcription start site (TSS) and stably pauses. GAF binding was previously implicated in *Hsp70* pausing, as a *Hsp70* transgene with a mutant GAGA element showed reduced pausing [28]. To test if GAF has a role in pausing on the endogenous *Hsp70* genes, we first treated cells with dsRNA targeting all isoforms of GAF, and reduced GAF levels to less than 10% of those in untreated or control cells treated with LacZ dsRNA (Fig. 1A). Chromatin-immunoprecipitation (ChIP) showed that GAF binding on the *Hsp70* promoters (-154bp from the TSS) decreased about 4-fold in NHS GAF-RNAi cells (Fig. 1B). We assayed the effect of GAF depletion on the paused polymerase present on NHS *Hsp70* using ChIP for the Pol II subunit, Rpb3. In untreated and LacZ-RNAi cells, Pol II levels were high at the 5’ end of *Hsp70* (+96bp from the TSS) and decreased in the gene body to near the levels on a non-transcribed (bkgd.) region (Fig. 1C). GAF knock-down resulted in 2-fold reduction in Pol II in the +96 region with no discernible change in the gene body (Fig. 1C). These results show that GAF has a role in maintaining the level of paused Pol II on the 5’ end of NHS *Hsp70*.

### Polymerase occupancy on many genes is GAF-dependent

**Fig 2:**
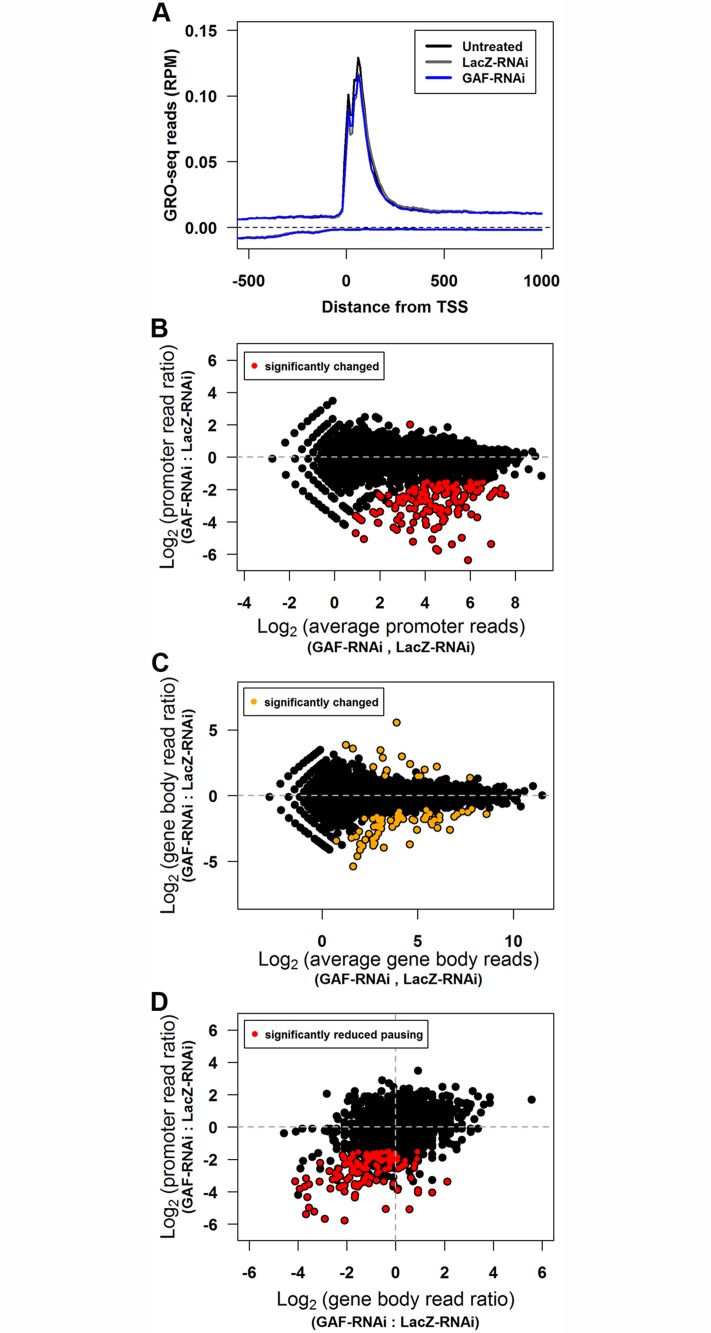
GAF knock-down reduces promoter-proximal polymerase on many genes. **(A)** The average GRO-seq reads (per million mapped reads) between 500bp upstream to 1000bp downstream for the TSS of all genes binned by 10bp. The reads from the sense strand are plotted above zero and the reads from the anti-sense strand are plotted below zero. **(B)** Promoter-proximal GRO-seq reads (100bp window with the most reads within 250bp of the TSS) of each gene for LacZ-RNAi and GAF-RNAi libraries plotted as the log_2_ ratio of GAF-RNAi to LacZ-RNAi reads is plotted on the y-axis and log_2_ of the average of LacZ-RNAi and GAF-RNAi reads on the x-axis. The regions with significant changes between the LacZ-RNAi and GAF-RNAi as determined by edgeR are colored red. **(C)** Gene body GRO-seq reads (500bp downstream of the TSS to the polyadenylation site) of each gene for LacZ-RNAi and GAF-RNAi libraries are plotted as in B. The regions with significant changes between the LacZ-RNAi and GAF-RNAi as determined by edgeR are colored orange. **(D)** The change in promoter-proximal and gene body reads represented as log_2_ of the GAF-RNAi to LacZ-RNAi ratio. The promoter regions with significant changes between the LacZ-RNAi and GAF-RNAi as determined by edgeR are colored red.

Previous ChIP-chip studies have shown that about 1,500 genes are bound by GAF in S2 cells and these genes are enriched for paused Pol II [4,25,26]. To test the role of GAF in transcription genome-wide, we performed GRO-seq in biological replicates of untreated, LacZ-RNAi, and GAF-RNAi cells to obtain the genome-wide distribution of transcriptionally-engaged polymerases [34]. GRO-seq maps polymerase by affinity purifying and sequencing nascent RNAs after bromo-UTP (BrUTP) incorporation in a nuclear run-on [34]. The density of sequence reads mapped within a region indicates the number of engaged polymerase in the cells from which the nuclei were isolated. In agreement with previous GRO-seq results in Drosophila [35–37] and genome-wide Pol II ChIP data [37,38], the average GRO-seq read profile for genes in each library displayed a peak of engaged polymerase on the 5’ end, and the average Pol II level was not changed by knock-down (Fig. 2A). To examine the polymerase distribution at individual genes, we quantified GRO-seq reads in the promoter-proximal and gene body regions for 9,452 non-overlapping genes [34,37]. We examined the transcription on each gene. Paused genes were defined as genes with significantly higher levels of engaged polymerase in the promoter region than the gene body (Fisher’s exact p-value <0.01). Transcriptionally active genes were defined as genes with significantly higher density of engaged polymerase in their gene body compared to 1% of mapped reads distributed uniformly across the Drosophila genome, the estimated level of background reads (p-value <0.01) [34]. We found that about half were significantly paused, and 60% of genes were actively transcribed. Notably, paused genes were highly enriched among those that were transcriptionally active (72% of transcribed genes were paused, and over 90% of paused genes were transcribed; Table 1).

**Table 1:** The number of paused and actively-transcribed genes for 9452 genes.

	All genes	Active genes	Inactive genes
All genes	9452	5682	3771
Paused genes	4558	4113	445
Non-paused genes	4895	1569	3326

The GRO-seq biological replicates were used to identify genes that significantly change between control and GAF-RNAi treatments. The biological replicates gave reproducible results: the promoter and gene body GRO-seq read counts for all biological replicates were highly correlated, with Pearson’s correlation coefficients (r) between 0.907–0.968 (S1 Table). Consistent with the similarity between the average GRO-seq read distribution across genes (Fig. 2A), the read counts for the combined replicates correlated well between the untreated and LacZ-RNAi libraries (promoters r = 0.984, gene bodies r = 0.997). We used edgeR to identify statistically significant changes with a false discovery rate corrected threshold q<0.01 in GRO-seq read counts separately in the promoter and gene body regions [39]. There were no genes with significantly different promoter read counts between the untreated and LacZ-RNAi libraries (S1A Fig), and only 5 genes had significantly different gene body read counts (S1B Fig, orange points). In contrast, there were 141 genes with significantly different read counts in the promoter-proximal region in GAF-RNAi and all but one was reduced (Fig. 2B, red points). The GAF-RNAi library had only 84 genes with gene body read levels significantly different from LacZ-RNAi. The majority of these were decreased (68 decreased and 16 increased) (Fig. 2C, orange points), and this bias for decreased reads following GAF-RNAi was highly statistically significant (p = 4.27x10^-9^, binomial test). These results support a role for GAF, beyond *Hsp70*, in maintaining levels of Pol II on the 5’ end of genes.

A reduction in recruitment and entry of Pol II into the pause site can lead to a decrease in elongating (gene body) polymerase. Indeed, recent studies have shown that disrupting initiation reduces both pausing and elongating polymerase [40,41]. Following GAF-RNAi, changes in polymerase density were more dramatic in the pause region than the gene body (S1E Fig and S1F Fig), and as a result many genes observed to have significant changes in the pause peak were not called statistically significant in the gene body by edgeR. We hypothesized that the lack of genome-wide statistical significance at many of these genes was because we were underpowered to identify smaller changes using only two biological replicates. To address this possibility, we asked whether genes that show a significant decrease in paused Pol II also show a significant bias for having a decrease in gene body Pol II. We found that genes with significantly reduced promoter GRO-seq reads upon GAF knock-down were also enriched for reductions in gene body reads (Fig. 2D, red points; p = 4.44x10^-16^, binomial test), demonstrating that, as a group, gene body Pol II decreased along with promoter proximal Pol II. These changes suggest that GAF plays a role early in the transcription cycle, allowing Pol II to initiate transcription and establish pausing at certain genes, which in turn influence the level of Pol II that progresses into the gene body.

### Genes with reduced promoter-proximal pausing have GAF-bound promoters

To assess if the effects on promoter-proximal polymerase levels are likely to be a direct effect of GAF knock down, we used ChIP-seq to analyze GAF binding sites and the sensitivity of GAF binding at each site to the reduced GAF protein levels in GAF-RNAi cells (S2A Fig). ChIP was performed with an affinity purified GAF antibody in both untreated and GAF-RNAi NHS S2 cells. The combined biological replicates of untreated control material identified 12,583 individual peaks and knock-down reduced binding on the large majority of sites (S2B Fig, S3 Table). The levels of control ChIP-seq reads within each peak correlated well with the previous ChIP-chip data [42] (S2C Fig, r = 0.887) and ChIP-qPCR for GAF at selected sites (S2D Fig, r = 0.718).

To evaluate if GAF is preferentially associated with promoter-proximal pausing, we first determined all the genes that have GAF ChIP-seq peaks within the promoter (within 500bp upstream of the TSS) and gene body. In our set of 9,452 non-overlapping genes, GAF was bound to 1,939 (S2 Table). The majority of these genes had at least one peak within their promoter (1,221; 63%). GAF-bound genes were significantly enriched for actively transcribed genes compared to all other genes (Fisher’s exact test, p < 2.2x10^-16^) and for paused genes (Fisher’s exact test, p < 2.2x10^-16^) or all other transcribed genes (Fisher’s exact test, p = 5.41x10^-5^), which is consistent with previous reports [4,25] (Table 2).

**Table 2:** The number of paused and actively-transcribed genes for 1939 GAF-bound genes.

	All genes	Active genes	Inactive genes
All genes	1939	1580	359
Paused genes	1484	1319	165
Non-paused genes	455	261	194

**Fig 3:**
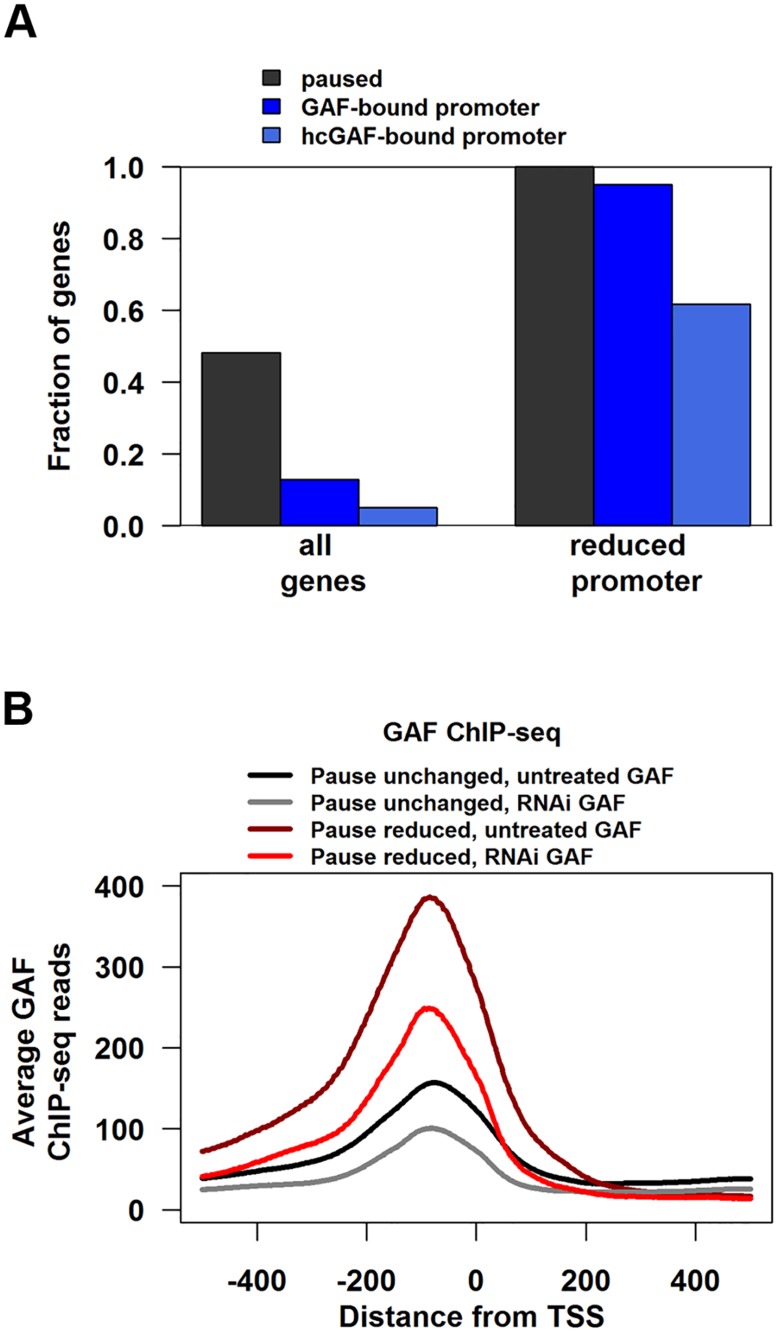
Genes with reduced pausing in GAF-RNAi are enriched for GAF-bound promoters. **(A)** Fraction of all genes or genes with significantly reduced promoter GRO-seq that are paused, have GAF-bound promoter, or high-confidence GAF peaks within their promoter. **(B)** The average GAF ChIP-seq reads from untreated (black and grey lines) or GAF-RNAi (maroon and red lines) cells between 500bp upstream to 500bp downstream for the TSS of paused genes with GAF-bound promoters separated into genes with significantly reduced promoter GRO-seq reads (Pause reduced) and all other paused genes with GAF-bound promoters (Paused unchanged).

The majority of genes with significantly reduced promoter GRO-seq reads in GAF RNAi-treated cells show GAF binding in untreated cells. Of the 140 genes with significant reduction in promoter GRO-seq reads between the GAF-RNAi and LacZ-RNAi libraries (reduced promoter), all of them were paused and 134 (95.7%) were bound by GAF (Fig. 3A). This suggests that changes in polymerase levels after GAF depletion are a primary effect of the knock-down and the effects of RNAi on levels of pausing are mediated through GAF acting locally at the gene, and not over a large chromatin domain.

Promoter-bound GAF cannot be the sole determinant for pausing because less than 14% of paused genes with GAF-bound promoters had significant reductions in promoter GRO-seq reads upon GAF knock-down. To investigate this further, we divided genes into two sets: paused genes with GAF-bound promoters that had significant reductions in promoter GRO-seq reads (hereafter referred to as Pause Reduced) and the other paused genes with GAF-bound promoters (hereafter referred to as Pause Unchanged). Then we looked for molecular signatures at GAF binding sites that correlated with the magnitude of pausing change after knock-down. The level of GAF binding on promoters was significantly higher in Pause Reduced genes than Pause Unchanged (Fig. 3B, black versus maroon line), even though GAF-binding was reduced by a similar fraction on Pause Reduced and Pause Unchanged promoters (Fig. 3B, gray versus black line and red versus maroon line).

The lower levels of GAF binding on Pause Unchanged genes raised the concern that these peaks could be an artifact and not bona fide GAF binding sites. To identify a high confidence subset of GAF peaks, we selected peaks with 2 additional criteria: they must overlap a peak region called in a dataset from an independent GAF antibody and they must contain a GAGA element. We used the modENCODE GAF ChIP-chip as the independent antibody dataset [42], and found that 9808 of our ChIP-seq peaks overlap with ChIP-chip enriched regions (S3 Table). GAGA elements were called using the position-weight matrix from the JASPAR database (Trl) [43] and 4,397 peaks had a GAGA element (defined using a p-value cutoff <1x10^-4^, S3 Table). Applying both criteria to our ChIP-seq peaks resulted in 3622 high-confidence GAF (hcGAF) peaks (S3 Table). Although hcGAF peaks were enriched on the Pause Reduced promoters as compared to Pause Unchanged promoters (Fig. 3A, Fisher’s exact test p = 4.542x10^-7^), 39% of Pause Unchanged promoters had hcGAF peaks, indicating many Pause Unchanged genes are likely truly bound by GAF.

### M1BP and Insulators are enriched on GAF-bound promoters of genes unaffected by GAF knock-down

**Fig 4:**
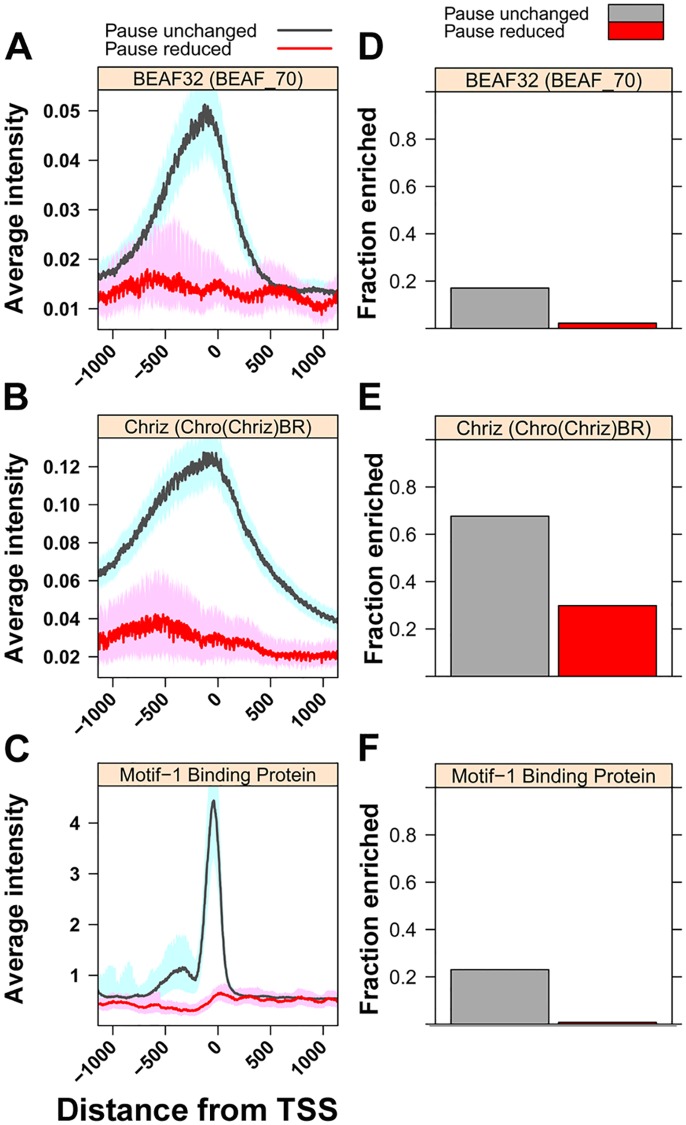
Levels of insulator-associated factors and Motif-1-binding protein are highest on unaffected genes. **(A)** The median intensity for the insulator protein BEAF32 (BEAF_70 ChIP-chip) 500bp upstream and downstream of the TSS of paused genes with GAF-bound promoters separated into genes with significantly reduced promoter GRO-seq reads (Pause reduced, red line) and all other paused genes with GAF-bound promoters (Pause unchanged, gray line). The shaded areas represent the 10% and 90% confidence intervals. **(B)** The same plot as in A for the chromodomain protein Chriz (Chro(Chriz)BR ChIP-chip). **(C)** The median ChIP-seq reads for the transcription factor Motif-1-binding protein ChIP-seq dataset, plotted the same as A. **(D)** Fraction of paused genes with GAF-bound promoters overlapping with regions of enrichment for BEAF32 in BEAF_70 ChIP-chip dataset within 500bp of their TSS. **(E)** The same plot as in D for Chriz in the Chro(Chriz)BR dataset. **(F)** The same plot as in D for Motif-1-binding protein ChIP-seq dataset on their promoter.

To identify the basis of the differential effects of GAF knock-down, we assessed whether other characteristics of paused genes with GAF-bound promoters correlate with the reduction in pausing. Individual labs and the modENCODE consortium have determined the genome-wide binding profiles for many chromatin-bound factors and histone modifications. We used this information to investigate if any of the factors with genome-wide data in S2 cells correlate with the GAF-RNAi effects on pausing (S3A Fig). Several factors were enriched on Pause Unchanged genes, but the most striking association was seen with BEAF32 [42,44], Chriz [42], and Motif-1-binding protein [45] binding levels (Fig. 4) and more modestly for other insulator factors (S3B-M Fig). Interestingly, BEAF, other insulators, and Chriz all colocalize at chromatin boundaries [46], and these proteins may insulate nearby promoters from the actions of locally bound GAF, making paused Pol II less sensitive to GAF knock-down.

Motif-1 Binding Protein (M1BP) is a transcription factor recently shown to be enriched on a set of paused genes, largely distinct from GAF-bound paused genes, and is believed to function analogously to GAF in Pol II pausing [45]. The striking enrichment of M1BP at Pause Unchanged genes suggests bound M1BP, and possibly other yet to be identified factors, provide functions redundant with GAF. We propose that pause-inducing redundant factors and insulator proteins conspire to render Pause Unchanged promoters insensitive to GAF.

### GAF normally keeps nucleosomes off promoters that show a GAF knock-down reduction in pausing

**Fig 5:**
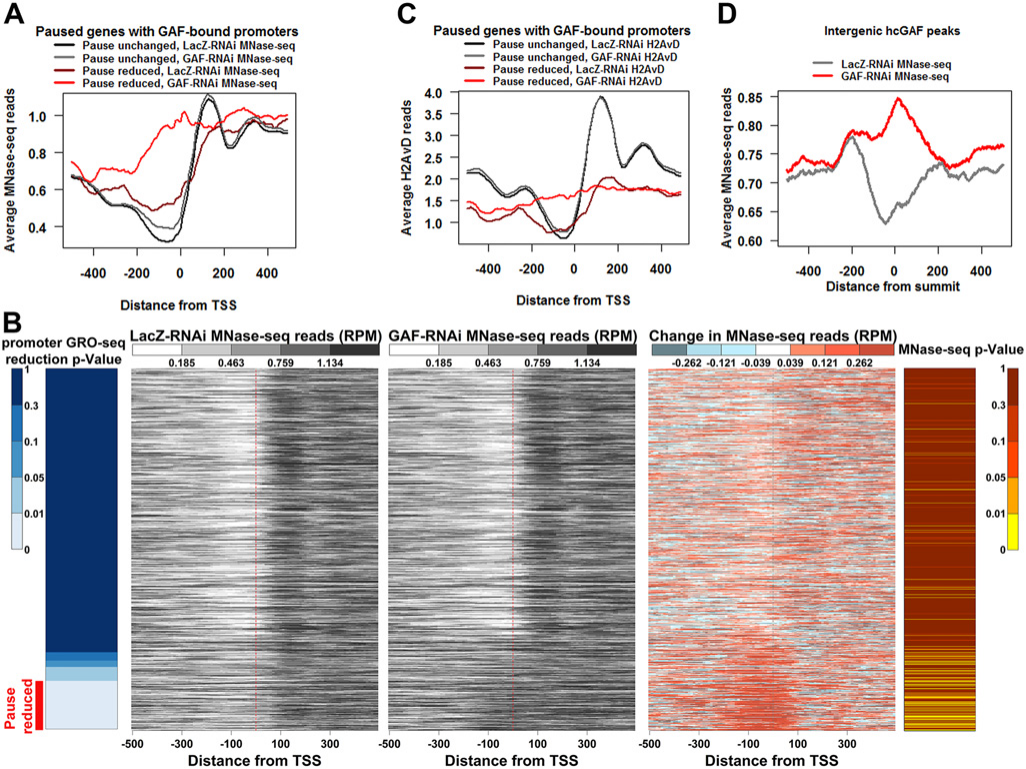
Promoters with reduced pausing fill-in with nucleosomes. **(A)** The average profile of LacZ-RNAi and GAF-RNAi MNase-seq reads 500bp upstream and downstream of the TSS of paused genes with GAF-bound promoters separated into genes with significantly reduced promoter GRO-seq reads (Pause reduced) and all other paused genes with GAF-bound promoters (Pause unchanged). **(B)** Heatmaps showing the LacZ-RNAi MNase-seq read level, GAF-RNAi MNase-seq read level, and the change in MNase-seq reads (GAF-RNAi subtracted from LacZ-RNAi) 500bp upstream and downstream from each TSS of paused genes with GAF-bound promoters arranged based on the significance of GRO-seq promoter read reduction in 10bp bins, as indicated by the left heatmap. The Pause reduced genes are indicated by the red bar at the bottom of the left heatmap. The p-values for increased MNase-seq reads from 100bp upstream to 50bp downstream of each TSS are indicated in the right heatmap. **(C)** The average profile of LacZ-RNAi and GAF-RNAi H2AvD reads 500bp upstream and downstream of the TSS of paused genes with GAF-bound promoters separated into Pause reduced genes and Pause unchanged genes. **(D)** The average profile of LacZ-RNAi and GAF-RNAi MNase-seq reads 500bp upstream and downstream of high confidence intergenic GAF peaks.

GAF has been shown to affect promoter accessibility through interactions with nucleosome remodelers [30,32,33,47]. To investigate whether the differential effects of GAF knock-down are due to changes genome-wide in promoter accessibility, we performed MNase-seq experiments in LacZ-RNAi and GAF-RNAi cells. Replicates within each treatment correlated well (S4 Table), and the combined replicates for both treatments had the same average level and expected distribution across genes grouped by transcriptional status (S4A-D Fig). We examined nucleosome-sized (120–180bp) MNase-seq reads across GAF-bound paused promoters. Pause Unchanged promoters had low levels of nucleosomes within the promoter increasing to a peak around 135bp downstream of the TSS in the control LacZ-RNAi condition, similar to that of typical transcribed genes (Fig. 5A, black line). Intriguingly, even with the normal levels of GAF in the LacZ-RNAi control, the Pause Reduced promoters had higher nucleosomes around their TSS and the nucleosomes were more disordered downstream (Fig. 5A, maroon line). When GAF was knocked-down, there was only a slight change in the distribution of nucleosomes in the Pause Unchanged promoters (Fig. 5A, gray line), but nucleosomes dramatically increased on the Pause Reduced promoters (Fig. 5A, red line). Heatmaps confirmed that individual promoters in each of these gene sets have changes that are consistent with the average profiles for each class (Fig. 5B). We used edgeR to determine the promoters with significant changes in MNase-seq reads and found that the Pause Reduced promoters were enriched for significantly increased MNase-seq reads (Fig. 5B, right panel). These results indicate that Pause Reduced promoters fill in with nucleosomes upon GAF knock-down.

The nucleosome-sized MNase-seq reads used may not necessarily be produced by a nucleosome. Therefore, to further validate these results, we immunoprecipitated the promoter-enriched histone variant H2AvD from the MNase-seq material. As expected, H2AvD levels were highest at the -1 and +1 nucleosomes bordering promoters of actively transcribed genes and these nucleosomes were not changed genome-wide by GAF knock-down (S4E-H Fig). Similar to the LacZ-RNAi MNase-seq results, the Pause Unchanged genes had higher levels of H2AvD and a more positioned +1 H2AvD-containing nucleosome than the Pause Reduced genes in the LacZ-RNAi control libraries (Fig. 5C, the black versus the maroon line). The Pause Unchanged H2AvD levels or position were not altered by GAF knock-down, but the Pause Reduced genes showed a dramatic increase in H2AvD in their promoter and the H2AvD levels were relatively even across the entire region (Fig. 5C the gray versus the red line, S5A Fig). Indeed, the Pause Reduced promoters were enriched for significant increases in H2AvD reads (S5A Fig, right panel). Thus, GAF is enabling these promoters to adopt a nucleosome-free conformation that may in turn allow polymerase to initiate, and indirectly, to establish a promoter-proximal pause state.

Recently, it was shown that the paused Pol II itself was important for preventing nucleosome encroachment into the promoter [48]. Therefore, the increases in promoter nucleosomes upon GAF knock-down could possibly be due to the reduction in paused polymerase by some GAF-dependent mechanism that is distinct from our proposed function of GAF in maintaining the nucleosome-free conformation. To test whether GAF can directly maintain nearby regions in a nucleosome-free conformation, we examined intergenic GAF-bound regions away from paused polymerases. Indeed, these regions had dramatically lower average levels of transcriptionally-engaged polymerase nearby (S6A Fig). We looked at 611 intergenic hcGAF peaks oriented based on the strand of the GAGA elements within them. We found the LacZ-RNAi control MNase-seq reads were higher on one side of the GAF peaks, suggesting a DNA sequence specific directionality to nucleosome placement (Fig. 5D, gray line). Moreover, MNase-seq reads dramatically increased in GAF knock-down library (Fig. 5D, red line), and this increase was most evident on the GAF peaks with largest ChIP-seq reduction in GAF binding upon GAF-RNAi (S5B Fig). Additionally, the levels of transcriptionally-engaged polymerase were similar between all hcGAF intergenic peaks and still dramatically lower than the promoter regions with paused Pol II, independent of reduction in GAF binding (S6B Fig). These results indicate GAF itself can direct the maintenance of a nucleosome-free region.

## Discussion

In this study, we examine the role of GAF in transcription and pausing genome-wide using GRO-seq to map transcriptionally-engaged polymerases in Drosophila S2 cells depleted for GAF. Almost all of the 140 paused genes with significant reductions in promoter-proximal polymerase levels upon GAF depletion had GAF bound in their promoters. This result indicates that these reductions were direct effects of GAF knock-down and GAF functioned locally at these genes to maintain paused Pol II levels. Moreover, we demonstrate that GAF has a prominent role in creating a chromatin accessible promoter for the recruitment and initiation of Pol II transcription. This opening of chromatin can be seen at GAF binding sites in promoters, but also at intergenic sites that are far from promoters. These results provide strong in vivo and genome-wide support for the hypothesis that GAF can mediate nucleosome displacement proximal to its binding site, as was proposed from in vitro studies that examined the interplay of GAF binding and an ATP-utilizing remodeler (NURF) on the *Hsp70* promoter [30].

### GAF-dependent nucleosome remodeling promotes pausing

Several points in the transcription cycle can be targeted to regulate the level of promoter-paused Pol II [1,49]. A TF may contribute to the recruitment of Pol II to the pause by acting at steps upstream of pausing to allow recruitment, initiation, and entry to the pause site (e.g. ERα) [50], or a TF can contribute more directly by creating or stabilizing the paused Pol II (e.g., Spt5/Spt4 and NELF) [51,52]. A TF can also accelerate the release of paused Pol II into productive elongation and thereby reduce the level of paused Pol II. The expectation for disrupting a factor that aids the steps in either recruitment or initiation is that the level of both paused Pol II and Pol II transcribing the gene body will decrease. For example, inhibition of the helicase TFIIH results in the decay of both paused Pol II and Pol II elongating across genes [40,41]. Our results indicate that GAF knock-down reduces levels of transcriptionally-engaged polymerase on the genes where promoter polymerase levels are significantly reduced. This suggests that these genes are dependent on GAF to allow recruitment and initiation providing the Pol II that will subsequently the pause, and thereby, indirectly helping to establish pausing.

Previous studies have shown that GAF can interact with several nucleosome remodelers and maintain promoters in a transcription-competent conformation, but these results have been limited to a few specific genes [31–33]. We found that nucleosome levels dramatically increase on the genes with significantly reduced promoter-proximal polymerase. Interestingly, we found that these genes had higher levels of nucleosomes on their promoter before GAF knock-down, suggesting there is already a competition between nucleosomes and paused Pol II on these promoters under normal conditions. Interpretation of the nucleosome increase upon GAF knock down at these genes is complicated by the recent report indicating that paused Pol II can keep some promoters open [48]. It is possible that GAF contributes directly to Pol II pausing and it is the loss of paused Pol II in GAF knockdowns that leads to increases in nucleosome occupancy. However, knockdown of GAF leads to dramatically increased nucleosome occupancy at GAF sites that are intergenic and away from paused promoters. Thus, GAF appears to be critical to opening chromatin structure at many sites independent of whether or not paused Pol II is present.

### TFs work together to regulate complex patterns of gene expression

Collectively, our analyses demonstrate how TFs can work together to regulate the expression of target genes. We find that only a subset of the paused genes with GAF-bound promoters had reductions in promoter polymerase levels upon GAF knockdown. GAF levels were higher on these genes, and this may reflect that stable binding of GAF is necessary to maintain the chromatin in an open conformation. Interestingly, the set of GAF bound genes whose promoter polymerase levels are insensitive to GAF knockdown are enriched for the transcription factor M1BP, the insulator protein BEAF, and the BEAF-interacting protein Chriz. M1BP was recently found to be enriched on paused genes that are mostly distinct from the group bound by GAF [45], suggesting that this TF might independently facilitate Pol II recruitment and initiation and partially compensate for the loss of GAF in the knockdown. In support of this, multiple mammalian TFs were shown to stimulate formation of paused Pol II without greatly affecting escape of paused Pol II into productive elongation [53,54]. Insulators might also act by unknown mechanisms to compensate for the loss of GAF, or the insulator may be blocking GAF’s action on promoters and allowing other factors like M1BP to independently cause Pol II to generate promoter-paused Pol II. Therefore, these results indicate that many of the genes lacking a significant effect following GAF knockdown are explained by the combinatorial patterns of factor binding and their interplay at the target promoter region.

### GAF can promote pausing through various interactions

GAF may function to specify pausing on bound genes by altering multiple steps in the transcription cycle. As we have shown, GAF can indirectly help to establish pausing by binding the promoter and maintaining nucleosome-free promoter regions that allow recruitment and initiation by Pol II. GAF may also have a direct role in initiation, as others have shown that GAF can itself act as an activator through its poly-glutamine domain [18,19] or may promote initiation through interactions with the TAF3 subunit of TFIID [55]. GAF can also interact with NELF to focus pausing in vitro on *Hsp70* more proximal to the TSS [56], although these changes in the position of the pause could not be picked up by the GRO-seq assay used here.

While GAF may act by more than one mechanism to generate and maintain paused Pol II, our results provide strong support for the hypothesis that GAF functions genome-wide to keep adjacent regions of chromatin nucleosome free. Our hypothesis is also consistent with previous reports that support a role of GAF as an anti-repressor for genes [29]. GAF might be simply competing with nucleosomes to promote chromatin accessibility; however, GAF is known to interact with several nucleosome remodelers: NURF, ISWI and BPAP, and displace adjacent nucleosomes to make DNA accessible regions [30–33]. We propose that the nucleosome landscape and Pol II occupancy at a subset of promoters is regulated by GAF’s recruitment of nucleosome remodelers and other factors, allowing Pol II entry and pausing.

## Materials and Methods

### RNAi

Drosophila S2 cells were grown in M3+BPYE+10% serum to a density between 3–5x10^6^cells/ml. After splitting to 1x10^6^cells/ml in serum-free M3 media (at least a 1:3 split), the desired volume of cells were mixed with 10μg/ml double-stranded RNA (dsRNA), incubated at 25°C for 45 minutes, and then, an equal volume of M3+BPYE+20% serum was added. After 5 days, the cells were harvested for the experiments. The dsRNAs were generated from a PCR template with T7 promoters on each end, targeting either a region conserved in all GAF isoforms or a region of B-galactosidase (LacZ) gene serving as a control.

GAF Forward: GAATTAATACGACTCACTATAGGGATGGTTATGTTGGCTGGCGTCAA

GAF-Reverse: GAATTAATACGACTCACTATAGGGATCTTTACGCGTGGTTTGCGT

LacZ-Forward: GAATTAATACGACTCACTATAGGGATCGTCAGTAGAAGAGCACCGAGT

LacZ-Reverse: GAATTAATACGACTCACTATAGGGAGAGATATCCTGCTGATGAAGC

### Chromatin immunoprecipitation (ChIP)

ChIP was performed as it was previously [57]. Briefly, after RNAi treatment, Drosophila S2 cell cultures were cross-linked for 2 minutes with formaldehyde at a 1% final concentration, and the cross-linking was quenched with glycine at a 125mM final concentration. The cell pellets were suspended to 1x10^8^cells/ml in sonication buffer (20mM Tris-Cl pH 8.0, 2mM EDTA, 0.5mM EGTA, 0.5% SDS, 0.5mM PMSF, protease inhibitor cocktail [Roche catalog no. 05 056 489 001]). The cells were sonicated 12 times for 20 seconds each time with a 1 minute rest in between at 4°C using a Bioruptor sonicator (Diagenode) on the highest setting.

The sonicated material was centrifuged at 20,000xg for 10 min at 4°C, and the supernatant was saved for the immunoprecipitation (IP). For each IP, 25μl of cleared sonication material was mixed with 1ml IP buffer (20mM Tris-Cl pH 8.0, 150mM NaCl, 2mM EDTA, 10% glycerol, 0.5% TritonX-100) with the antisera (10μl affinity purified Anti-GAF antibody [58] or 4μl of rabbit anti-Rpb3 antisera [59]) at 4°C overnight. For ChIP-qPCR, a standard curve of 10%, 1%, 0.1%, and 0.01% of input DNA and the immunoprecipitated DNA were quantified using a Roche LightCycler 480, and the standard curve was used to determine the amount of DNA immunoprecipitated.

For the ChIP-seq, two replicates of chromatin immunoprecipitation (ChIP) were carried out for each condition, as previously described [60], and sequenced using Illumina GAIIx sequencer.

### GRO-seq

GRO-seq libraries were constructed using previous methods [57]. Briefly, nuclei were isolated from RNAi-treated cells. Each nuclear run-on was performed for 10 minutes at 30°C with 2x10^7^ nuclei in run-on buffer (10mM Tris-Cl pH 8.0, 5mM MgCl_2_, 300mM KCl, 500μM ATP, 500uM GTP, 2μM CTP (cold), 1mCi/ml ^32^P-CTP (100μCi/ run-on), 500μM Br-UTP, 0.4 units Superase-In, 1mM DTT, 40 units Superase-In (Ambion), 0.6% N-lauroyl-sarcosine), and stopped with 1.5ml Trizol and 200μl chloroform. After extraction with acid phenol:chloroform and chloroform, the precipitated RNAs were resuspended in 20μl DEPC-treated ddH_2_O, and hydrolyzed in 200mM NaOH on ice for 18 minutes. The hydrolyzed RNAs purified by three bead bindings to Anti-Br-dUTP beads (blocked with 0.1% polyvinylpyrrolidone and 1μg/ml BSA). The beads were washed once with 500μl binding buffer, once with 500μl Low salt buffer (0.2x SSPE, 1mM EDTA, 0.05% Tween-20), once with 500μl High salt wash (0.25x SSPE, 1mM EDTA, 137.5mM NaCl, 0.05% Tween-20), and twice with 500μl TET wash (10mM Tris-Cl pH 7.5, 1mM EDTA, 0.05% Tween-20). After elution with elution buffer (50mM Tris-Cl pH 7.5, 150mM NaCl, 1mM EDTA, 0.1% SDS, 20mM DTT), the precipitated RNAs were resuspended in 20μl DEPC-treated ddH_2_0. After the first bead binding, RNAs are treated with T4 polynucleotide kinase (PNK) without ATP to create a 3’ hydroxyl group. Illumina linkers were added using polyadenylation with E. coli polyA polymerase and reverse transcription from a poly(dT)-3’linker covalently attached to the 5’ linker with a 18 carbon spacer, as previously used [37,61]. Each library was made in biological replicates, and bar-coded using specific reverse transcription primers (INOO3: 5’-pTAGAGATCGTCGGACTGTAGAACTCT-iSp18-CAAGCAGAAGACGGCATACGATTTTTTTTTTTTTTTTTTTTVN, INOO4: 5’-pTGATGATCGTCGGACTGTAGAACTCT-iSp18-CAAGCAGAAGACGGCATACGATTTTTTTTTTTTTTTTTTTTVN). The cDNA was circularized using Circligase (Epicentre catalog # CL4111K) to connect the 5’ linker to the 5’ end of the cDNA. After PCR amplification, the libraries were gel purified away from the primers, each replicate library was combined in equal amounts, and sequenced for 50 bases on one lane of an Illumina GIIAx sequencer.

### MNase-seq

MNase-seq material was created similar to previous studies [48]. Briefly, RNAi-treated cells were cross-linked identically to the ChIP protocol. Nuclei were isolated from the cross-linked cells, and digested so that 80% of DNA was mononucleosome size. For H2AvD nucleosomes, 75ul of material was immunoprecipitated with 4ul Anti-H2AvD antisera (Glaser lab). After reversal of cross-links, Illumina paired-end TruSeq adapter were ligated to 50ng of DNA using standard protocols, and amplified for 10 cycles. The DNA was size selected for inserts between 80–280bp in length, and paired-end sequencing for 50 bases (each end) was performed on an Illumina Hi-seq sequencer. Reads were aligned to the Drosophila dm3 genome using bowtie2 (—no-mixed—no-discordant). Mononucleosome-sized reads between 120 and 180 bases were selected computationally. The heatmaps and composite profiles used the whole reads mapped to the genome, and the centers of each read were used to calculate significance of changes in read counts with edgeR.

### Peak calling

MACS1.4 was used to initially call peaks using the combined replicate data compared against pre-immune IP data (MACS parameters: effective genome size = 1.65e+08, band width = 150, model fold = 10,10000, p-value cutoff = 1.00e-04, Range for calculating regional lambda is: 1000 bps and 10000 bps). Closely clustered subpeaks, within broad regions of MACS-identified peaks, were deconvoluted by using the Subpeaks tool contributed to MACS [62].

### Designation of high-confidence GAF (hcGAF) peaks

We defined high-confidence GAF peaks based on overlap with peaks in an independent GAF dataset and the presence of a GAGA element within our peak. We selected our untreated ChIP-seq peaks that overlapped with the “Regions_of_sig_enrichment” in the modENCODE GAF ChIP-chip GFF3 file [42]. We identified GAGA elements with FIMO (p-value threshold 1x10^-4^) using the JASPAR Trl motif [43,63].

### Data analysis

All mapping, quantification, and transcriptional status determinations were performed as in previous studies [34]. The reads for each treatment were normalized to total mapped reads. To validate that GAF-RNAi was not changing the amount of transcriptionally-engaged Pol II genome-wide, we also used the total number of mapped Pol I and Pol III reads to normalize with little change in results.

We identified paused genes based on higher levels of engaged polymerase in the promoter region than the gene body compared to the number of reads in each region when the reads are uniformly distributed across the gene (Fisher’s exact test p-value <0.01). Transcriptional activity was defined exactly as previously [34]. We calculated the probability that the observed gene body read counts were generated from a Poisson distribution, with a mean equal to the observed background density (1% of mapped reads uniformly distributed) times the number of mappable bases in the gene body. Genes with more reads than expected under the background null model (p< 0.01) were considered transcriptionally active.

Regions with significant changes in GRO-seq reads between Untreated or LacZ-RNAi and GAF-RNAi were called using the edgeR package (v.1.4.1) setting a false discovery rate threshold of q = 0.01 [39]. The MNase-seq and H2AvD read centers between 100bp upstream and 50bp downstream of each TSS or 100bp around each intergenic hcGAF peak were used in edgeR to call significantly changed promoters.

The Fisher’s exact test showing GAF-bound genes are enriched for transcriptional activity compared the number of transcriptionally active genes for GAF-bound genes (1580 out of 1939) to all genes (4102 out of 7513). Because GAF-bound genes are dramatically enriched for actively transcribed genes, the Fisher’s exact test showing GAF-bound genes are enriched for pausing compared the number of paused genes for GAF-bound genes (1484 out of 1939) to all other genes (3074 out of 7519). The Fisher’s exact test showing Pause Reduced promoters are more likely to have hcGAF peaks than Pause Unchanged promoters compared the number of hcGAF-bound Pause Reduced promoters (87 out of 134) to hcGAF-bound Pause Unchanged promoters (320 out of 1078).

A binomial test was used to show that genes with statistically significant gene body changes are more likely to be down-regulated than up-regulated, assuming that gene body changes are equally likely to be up- or down-regulated. A binomial test was also used to test whether there is a correlation between changes at the promoter and in the gene body, at genes with significant changes in promoter read counts. To address this, we asked whether genes with significantly reduced promoter GRO-seq reads are more likely to have a positive or negative gene-body log fold-change than expected by chance, under an equal probability for reduced and increased reads.

### Factor occupancy and intensity quantifications

Quantifications used in the graphs were obtained from the genome-wide datasets for factors/modifications created from S2 cells. For the Pearson correlation in S3 Fig, the level of various factors, histones, and histone modifications was calculated within 500bp of each TSS for GAF-bound promoters and compared to the ratio of promoter GRO-seq reads in GAF-RNAi and LacZ-RNAi libraries. For composite profiles, factor intensity was calculated at each base, unless otherwise indicated. The composite profiles in Fig. 4, S3 Fig, and S4 Fig are the median from 1000 samplings of 10% of genes, and the shaded areas in Fig. 4 and S3 Fig indicate the 10% and 90% confidence intervals. For the enrichment barplots in Fig. 4 and S3 Fig, the GFF3 files for each modENCODE factor and MACS peak bed files for each ChIP-seq datasets were used to identify enriched regions in the ChIP datasets that overlap the TSS.

### Accession numbers

The genomic data in this work is deposited in the Gene Expression Omnibus under the accession numbers: GSE58957 and GSE40646.

## Supporting Information

S1 Fig Untreated and LacZ-RNAi GRO-seq reads agree. **(A)** Promoter-proximal GRO-seq reads of each gene for LacZ-RNAi and Untreated libraries plotted in an MA plot. The log_2_ ratio of Untreated to LacZ-RNAi reads is plotted on the y-axis and log_2_ of the average of LacZ-RNAi and Untreated reads on the x-axis. There were no significantly changed regions. **(B)** Gene body GRO-seq reads of each gene for LacZ-RNAi and Untreated libraries are plotted as in B. Genes with significantly different reads between the two libraries are colored orange. One gene with significantly different reads did not have any reads in the Untreated library and could not be plotted. **(C)** Promoter-proximal GRO-seq reads of each gene for GAF-RNAi and Untreated libraries plotted as in A. Genes with significantly different reads between the two libraries are colored red. **(D)** Gene body GRO-seq reads of each gene for GAF-RNAi and Untreated libraries are plotted as in C. **(E)** The average LacZ-RNAi (black or maroon) and GAF-RNAi (gray or red) GRO-seq reads (per million mapped reads) binned by 10bp between 200bp upstream to 1000bp downstream of the TSS of the paused genes with GAF-bound promoters, separated into Pause unchanged (black or gray) and Pause reduced (maroon or red). **(F)** Boxplot showing log_2_ of ratio of GAF-RNAi to LacZ-RNAi promoter and gene body GRO-seq reads for Pause reduced and Pause unchanged genes.
(TIF)

S2 Fig GAF binding at GAF ChIP-seq peaks. **(A)** Western blot of whole cell extracts from Untreated and GAF-RNAi (GAF) cells for GAF and a loading control, TFIIS (1 is equivalent to 1x10^6^ cells). **(B)** A plot showing log_10_ of the peak intensity for untreated ChIP-seq data on the x-axis and log_2_ of the ratio of peak intensity between the GAF-RNAi and untreated ChIP-seq data on the y-axis for all peaks (12582). The colors indicate the number of data points within the area, created using the hexbin R package. **(C)** A plot comparing the GAF ChIP-chip intensities from the modENCODE project and the GAF ChIP-seq intensity in untreated cells. **(D)** A plot comparing the signal for GAF ChIP-qPCR and the ChIP-seq intensity in untreated cells at select GAF peaks. The Pearson’s correlation coefficient is indicated in the top-left of the panels.
(TIF)

S3 Fig Levels of insulator-associated factors are higher on Pause unchanged genes. **(A)** Heatmap showing the Pearson’s correlation coefficients comparing the change in promoter GRO-seq reads and the level of various factors, histones, and histone modifications at GAF-bound promoters. **(B-G)** The median intensity (ChIP-chip or ChIP-seq reads) 500bp upstream and downstream of the TSS for paused genes with GAF-bound promoters separated into genes with significantly reduced promoter GRO-seq reads (Pause reduced, red line) and all other paused genes with GAF-bound promoters (Pause unchanged, gray line) for the BEAF_HB ChIP-chip [42], BEAF32 ChIP-seq [44], and Chro(Chriz)WR ChIP-chip datasets [42], CP190_HB ChIP-chip [42],CP190_VC [42], and CTCF_VC [42] plotted as in Fig. 4. The shaded areas represent the 10% and 90% confidence intervals. **(H-M)** Fraction of Pause reduced and Pause unchanged promoters overlapping with region of enrichment in the same datasets as in B-G within 500bp of their TSS, plotted as in Fig. 4.
(TIF)

S4 Fig GAF-RNAi does not change genome-wide nucleosome distribution. **(A-D)** The average profile of LacZ-RNAi (gray line) and GAF-RNAi (red line) MNase-seq reads 1Kb upstream and downstream of the TSS for all genes **(A)**, paused genes **(B)**, LacZ-RNAi pausing p-value < 0.01), non-paused genes (**C)**, LacZ-RNAi pausing p-value > 0.01), silent genes (**D**, LacZ-RNAi GRO-seq gene body reads < 1). **(E-H)** The average profile of LacZ-RNAi and GAF-RNAi H2AvD reads 1Kb upstream and downstream of the TSS for the same gene groups.
(TIF)

S5 Fig Nucleosomes fill into intergenic GAF binding sites. **(A)** Heatmaps showing the p-value for the GRO-seq promoter read reduction upon GAF-RNAi (left panel), LacZ-RNAi H2AvD read level (second panel), GAF-RNAi H2AvD read level (third panel), the change in H2AvD reads (GAF-RNAi subtracted from LacZ-RNAi, fourth panel), and p-values for an increase in H2AvD reads (100bp upstream to 50bp downstream of each TSS) upon GAF-RNAi (right panel), as in Fig. 5B. **(B)** Heatmaps showing the same data as in A for intergenic hcGAF peaks arranged based on the reduction in GAF binding upon GAF-RNAi, as indicated by the left heatmap. The p-values for increased MNase-seq reads within 100bp upstream and downstream of each peak summit are indicated in the right heatmap.
(TIF)

S6 Fig Intergenic GAF sites have low levels of transcribing polymerase. **(A)** Boxplot showing log_10_ of the total LacZ-RNAi GRO-seq read within 200bp of promoters, gene body, and intergenic GAF peaks. **(B)** Boxplot showing log_10_ of the total LacZ-RNAi GRO-seq read within 200bp of intergenic hcGAF peaks, separated into quintiles based on reduction in GAF binding upon GAF-RNAi.
(TIF)

S1 Table GRO-seq replicates correlate well. A file with the total GRO-seq reads mapped to genes transcribed by RNA polymerase II, RNA polymerase I, the 5S rRNA, tRNAs, RNA polymerase III, and total reads mapped. The Pearson’s correlation coefficients GRO-seq read counts in promoter and gene body regions for 9452 genes.
(XLS)

S2 Table Summary of GRO-seq and GAF ChIP-seq binding for 9452 genes. The table contains 9452 genes unambiguous promoters and gene body regions (as used previously for classifying Paused genes [37]. Column 1: transcript name, Column 2: chromosome, Column 3 and 4: transcript borders (start and end), Column 5: strand, Column 6–17: GRO-seq reads counts and read counts normalized per million mapped Pol II reads in the promoter or the gene body for the combined untreated, LacZ-RNAi, and GAF-RNAi, Column 18–23: edgeR p-value for promoter or gene body read changes between untreated and LacZ-RNAi, untreated and GAF-RNAi, and LacZ-RNA and GAF-RNAi, Column 24: location of GAF ChIP-seq relative to the gene, Column 25: pausing index (ratio of promoter read density to gene body read density, a value of -1 indicates that there are no reads in the gene body) for LacZ-RNAi, Column 25: p-value for pausing determination in LacZ-RNAi (see methods), Column 26: p-value for active transcription in gene body for LacZ-RNAi (see methods section).
(XLS)

S3 Table GAF ChIP-seq peaks called by MACS. The columns show the chromosome, start, end, and summit, peak name, GAF level in untreated and GAF-RNAi ChIP-seq, whether the peaks are also called in the individual replicates, overlap with “region of significant enrichment” in untreated modENCODE ChIP-chip GFF3 file, number of GAGA elements in peak region, strand location for the majority of GAGA elements within the peak region, high-confidence call, location relative to genes, and total GRO-seq reads in LacZ-RNAi libraries within 100bp of the peak summit.
(XLS)

S4 Table MNase-seq and H2AvD replicate correlate well. The total sequenced paired-end reads, reads that passed filters, mapped reads and 120–180bp mapped reads for LacZ-RNAi and GAF-RNAi MNase and H2AvD triplicate libraries. The Pearson’s correlations are for read counts in 500bp bins across the genome for MNase-seq and H2AvD replicates.
(XLS)
