# Supplementary figures and images for: GAGA Factor Maintains Nucleosome-Free Regions and Has a Role in RNA Polymerase II Recruitment to Promoters
Source: PLoS Genet. 2015 Mar 27;11(3):e1005108. doi: 10.1371/journal.pgen.1005108 (PMC4376892; doi:10.1371/journal.pgen.1005108)

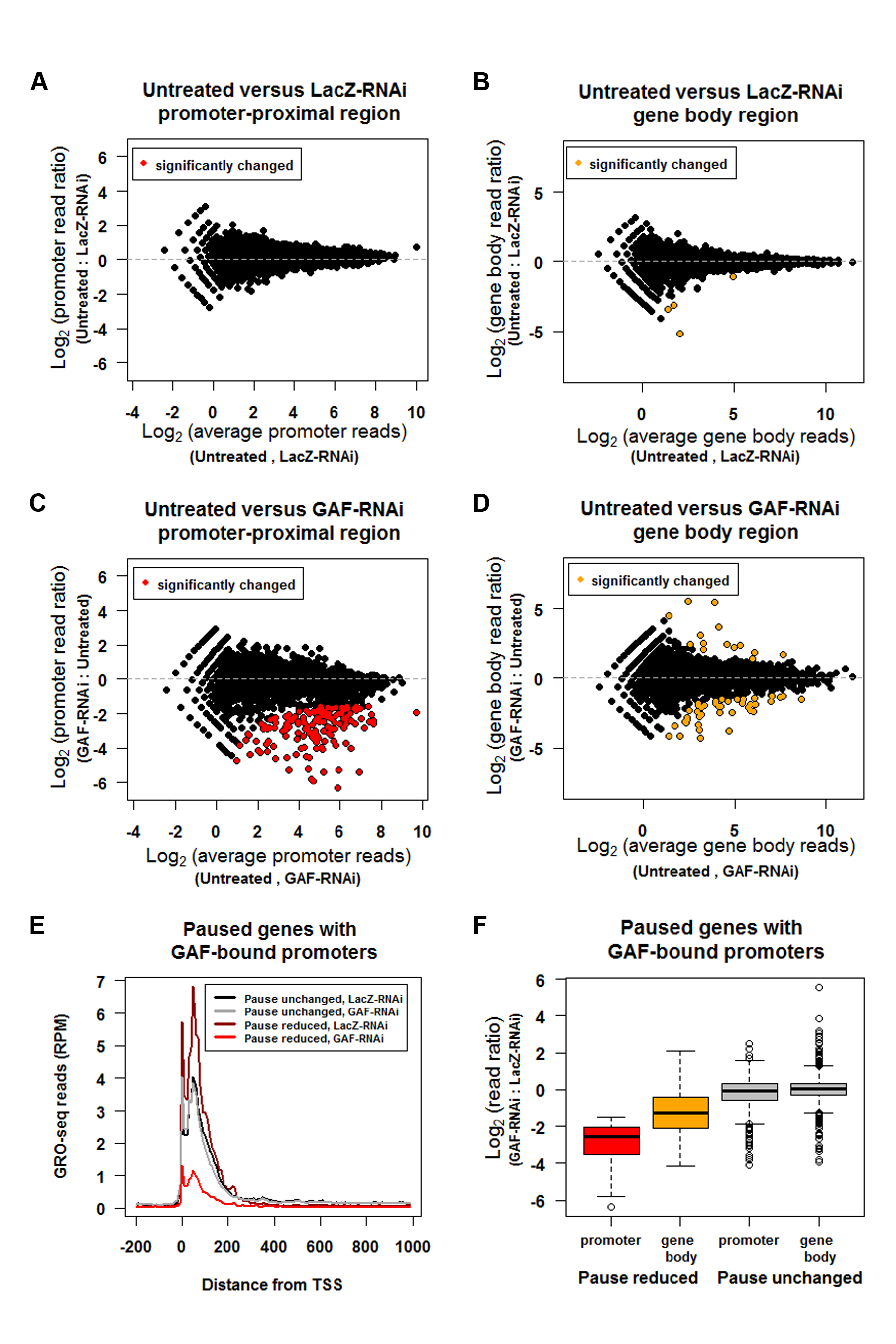

Supplement: S1 Fig — (A) Promoter-proximal GRO-seq reads of each gene for LacZ-RNAi and Untreated libraries plotted in an MA plot. The log2 ratio of Untreated to LacZ-RNAi reads is plotted on the y-axis and log2 of the average of LacZ-RNAi and Untreated reads on the x-axis. There were no significantly changed regions. (B) Gene body GRO-seq reads of each gene for LacZ-RNAi and Untreated libraries are plotted as in B. Genes with significantly different reads between the two libraries are colored orange. One gene with significantly different reads did not have any reads in the Untreated library and could not be plotted. (C) Promoter-proximal GRO-seq reads of each gene for GAF-RNAi and Untreated libraries plotted as in A. Genes with significantly different reads between the two libraries are colored red. (D) Gene body GRO-seq reads of each gene for GAF-RNAi and Untreated libraries are plotted as in C. (E) The average LacZ-RNAi (black or maroon) and GAF-RNAi (gray or red) GRO-seq reads (per million mapped reads) binned by 10bp between 200bp upstream to 1000bp downstream of the TSS of the paused genes with GAF-bound promoters, separated into Pause unchanged (black or gray) and Pause reduced (maroon or red). (F) Boxplot showing log2 of ratio of GAF-RNAi to LacZ-RNAi promoter and gene body GRO-seq reads for Pause reduced and Pause unchanged genes. (TIF) [file pgen.1005108.s001.tif]

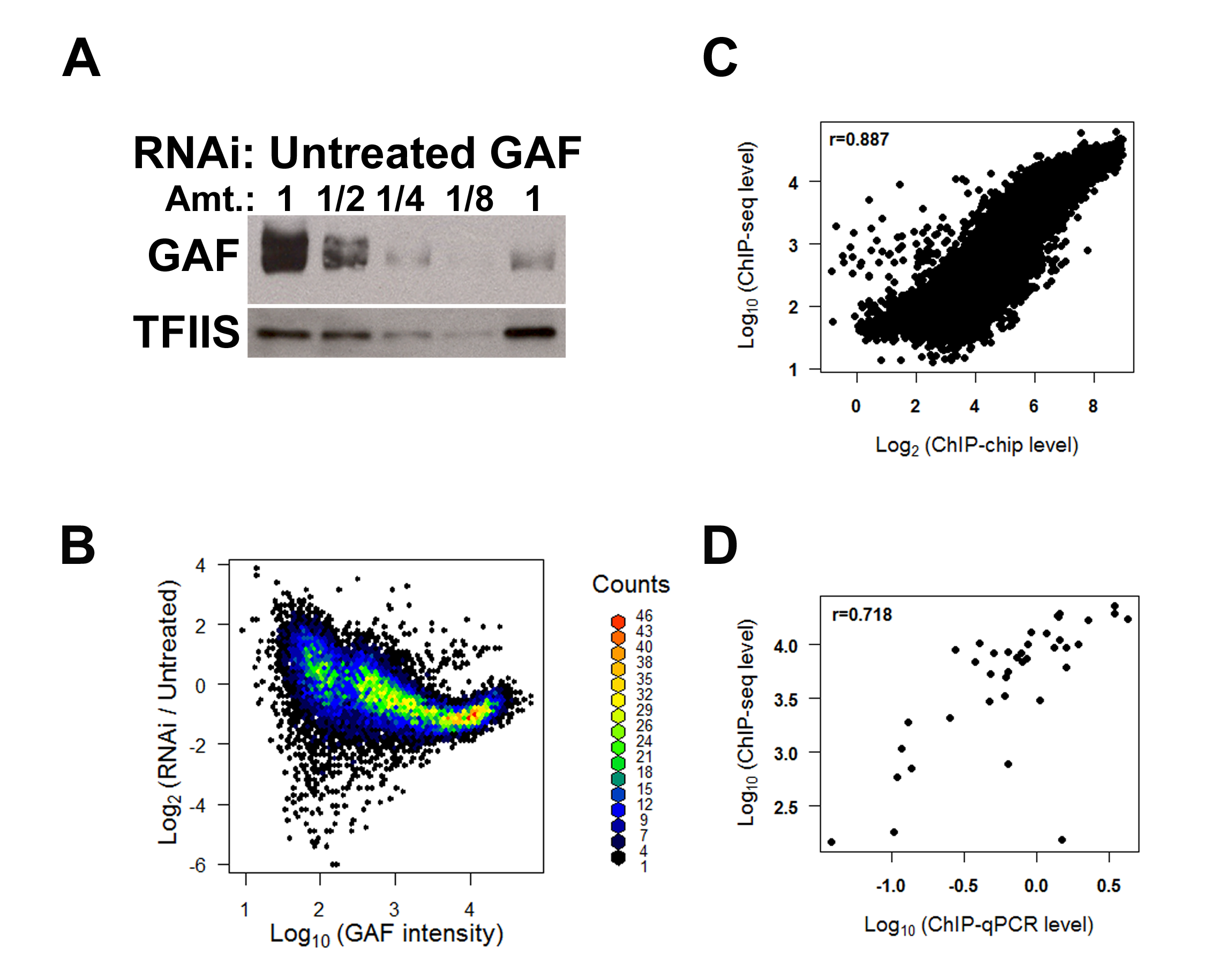

Supplement: S2 Fig — (A) Western blot of whole cell extracts from Untreated and GAF-RNAi (GAF) cells for GAF and a loading control, TFIIS (1 is equivalent to 1x10^6^ cells). (B) A plot showing log10 of the peak intensity for untreated ChIP-seq data on the x-axis and log2 of the ratio of peak intensity between the GAF-RNAi and untreated ChIP-seq data on the y-axis for all peaks (12582). The colors indicate the number of data points within the area, created using the hexbin R package. (C) A plot comparing the GAF ChIP-chip intensities from the modENCODE project and the GAF ChIP-seq intensity in untreated cells. (D) A plot comparing the signal for GAF ChIP-qPCR and the ChIP-seq intensity in untreated cells at select GAF peaks. The Pearson’s correlation coefficient is indicated in the top-left of the panels. (TIF) [file pgen.1005108.s002.tif]

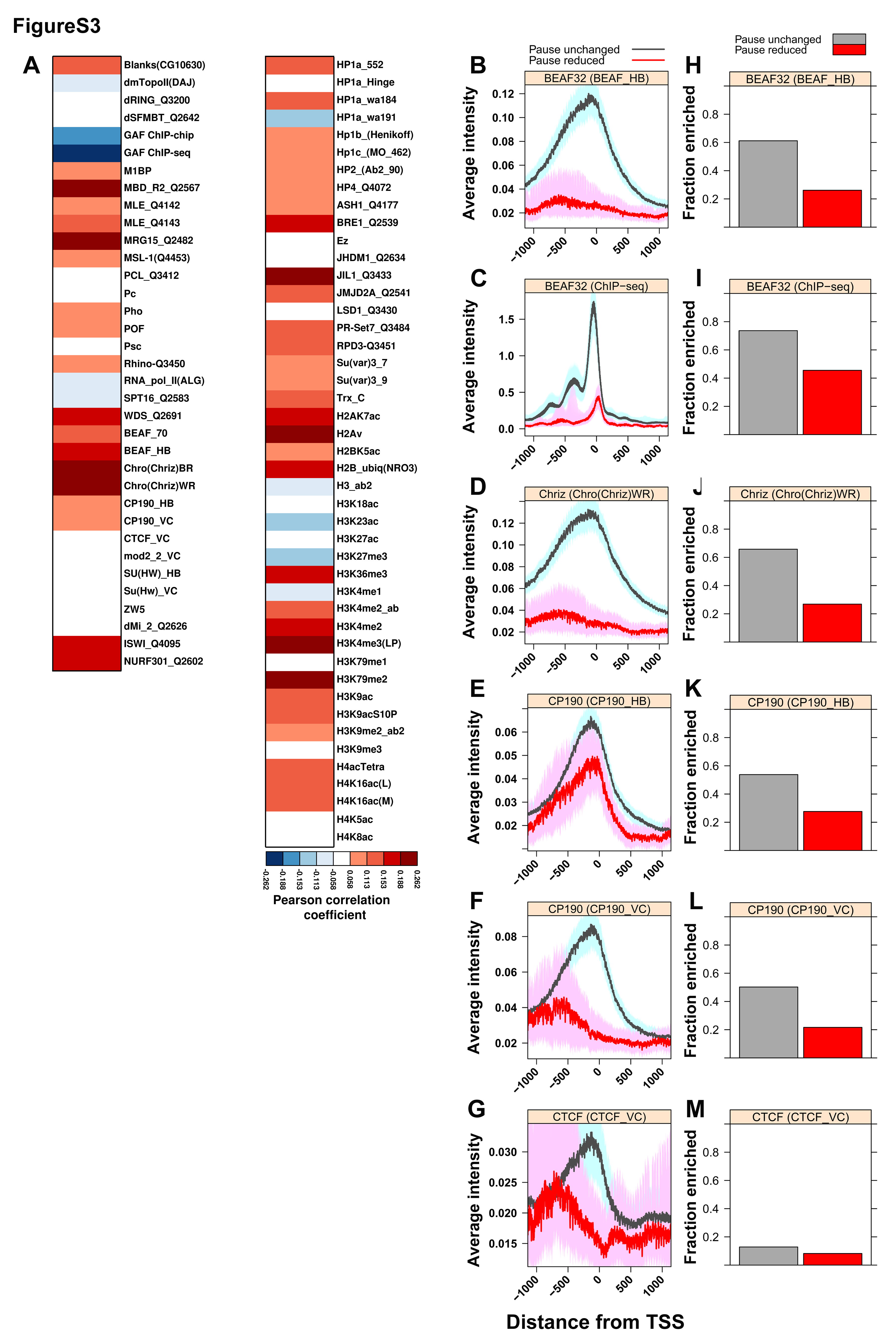

Supplement: S3 Fig — (A) Heatmap showing the Pearson’s correlation coefficients comparing the change in promoter GRO-seq reads and the level of various factors, histones, and histone modifications at GAF-bound promoters. (B-G) The median intensity (ChIP-chip or ChIP-seq reads) 500bp upstream and downstream of the TSS for paused genes with GAF-bound promoters separated into genes with significantly reduced promoter GRO-seq reads (Pause reduced, red line) and all other paused genes with GAF-bound promoters (Pause unchanged, gray line) for the BEAF_HB ChIP-chip [42], BEAF32 ChIP-seq [44], and Chro(Chriz)WR ChIP-chip datasets [42], CP190_HB ChIP-chip [42],CP190_VC [42], and CTCF_VC [42] plotted as in Fig. 4. The shaded areas represent the 10% and 90% confidence intervals. (H-M) Fraction of Pause reduced and Pause unchanged promoters overlapping with region of enrichment in the same datasets as in B-G within 500bp of their TSS, plotted as in Fig. 4. (TIF) [file pgen.1005108.s003.tif]

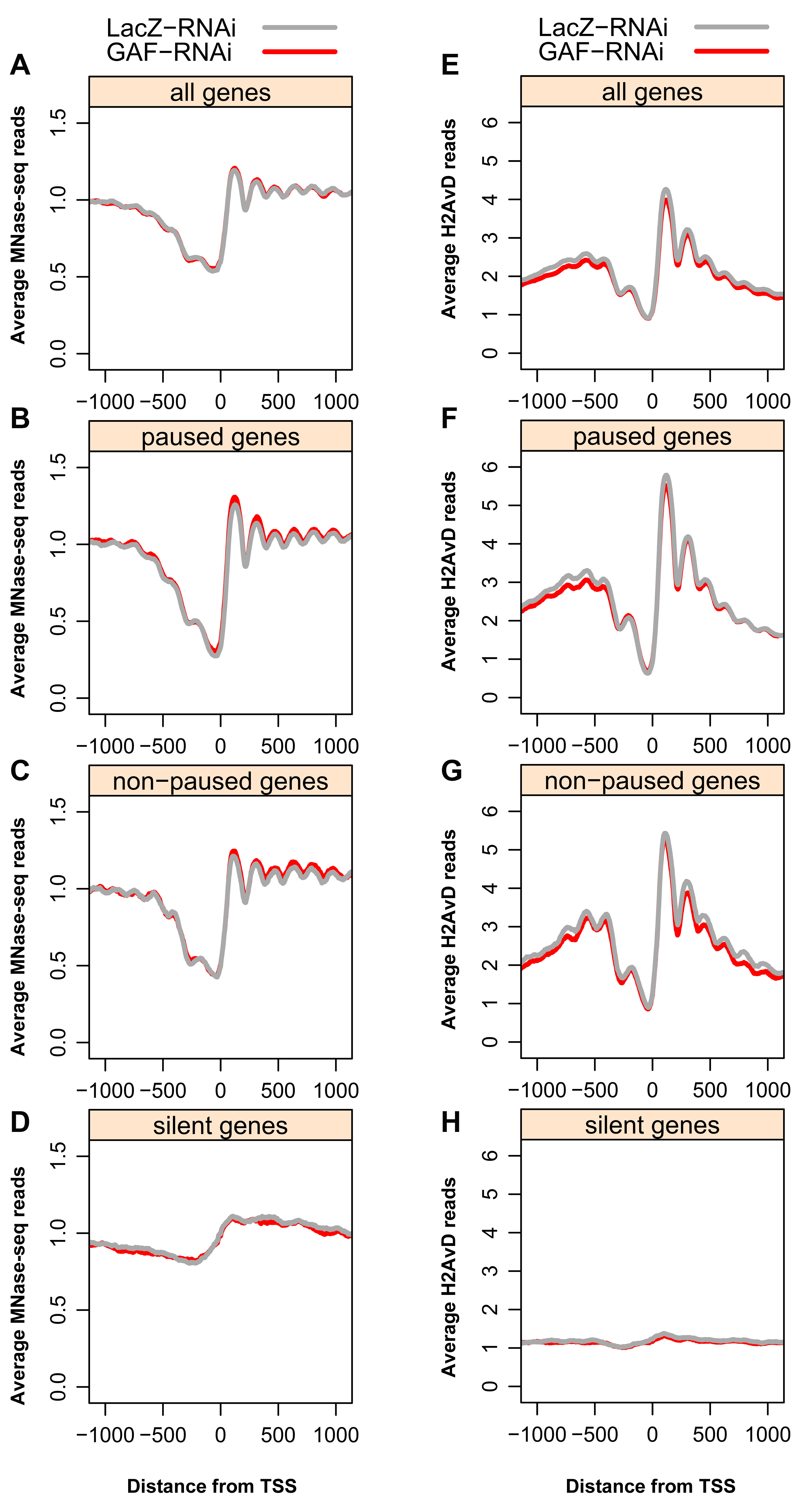

Supplement: S4 Fig — (A-D) The average profile of LacZ-RNAi (gray line) and GAF-RNAi (red line) MNase-seq reads 1Kb upstream and downstream of the TSS for all genes (A), paused genes (B), LacZ-RNAi pausing p-value < 0.01), non-paused genes (C), LacZ-RNAi pausing p-value > 0.01), silent genes (D, LacZ-RNAi GRO-seq gene body reads < 1). (E-H) The average profile of LacZ-RNAi and GAF-RNAi H2AvD reads 1Kb upstream and downstream of the TSS for the same gene groups. (TIF) [file pgen.1005108.s004.tif]

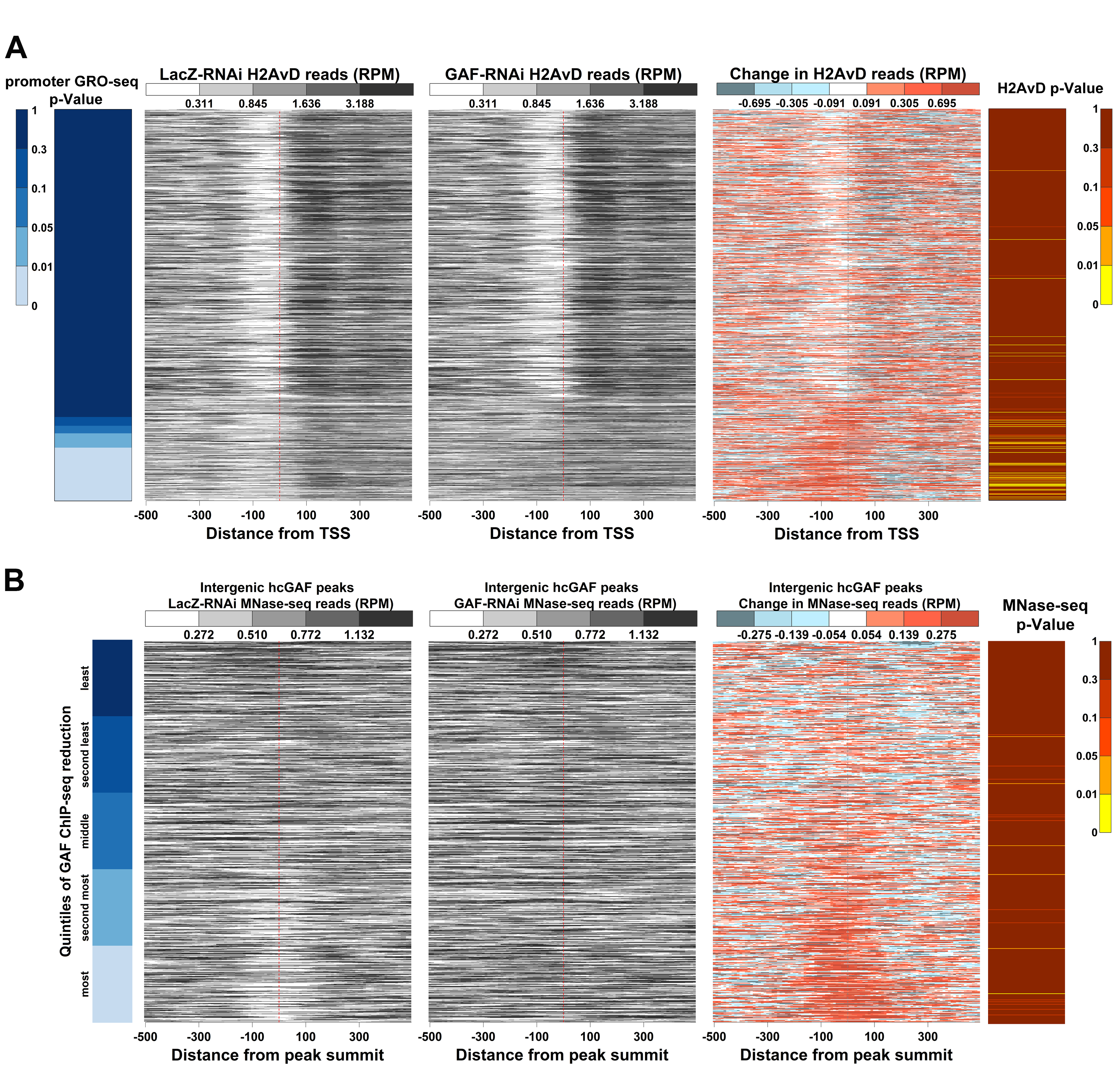

Supplement: S5 Fig — (A) Heatmaps showing the p-value for the GRO-seq promoter read reduction upon GAF-RNAi (left panel), LacZ-RNAi H2AvD read level (second panel), GAF-RNAi H2AvD read level (third panel), the change in H2AvD reads (GAF-RNAi subtracted from LacZ-RNAi, fourth panel), and p-values for an increase in H2AvD reads (100bp upstream to 50bp downstream of each TSS) upon GAF-RNAi (right panel), as in Fig. 5B. (B) Heatmaps showing the same data as in A for intergenic hcGAF peaks arranged based on the reduction in GAF binding upon GAF-RNAi, as indicated by the left heatmap. The p-values for increased MNase-seq reads within 100bp upstream and downstream of each peak summit are indicated in the right heatmap. (TIF) [file pgen.1005108.s005.tif]

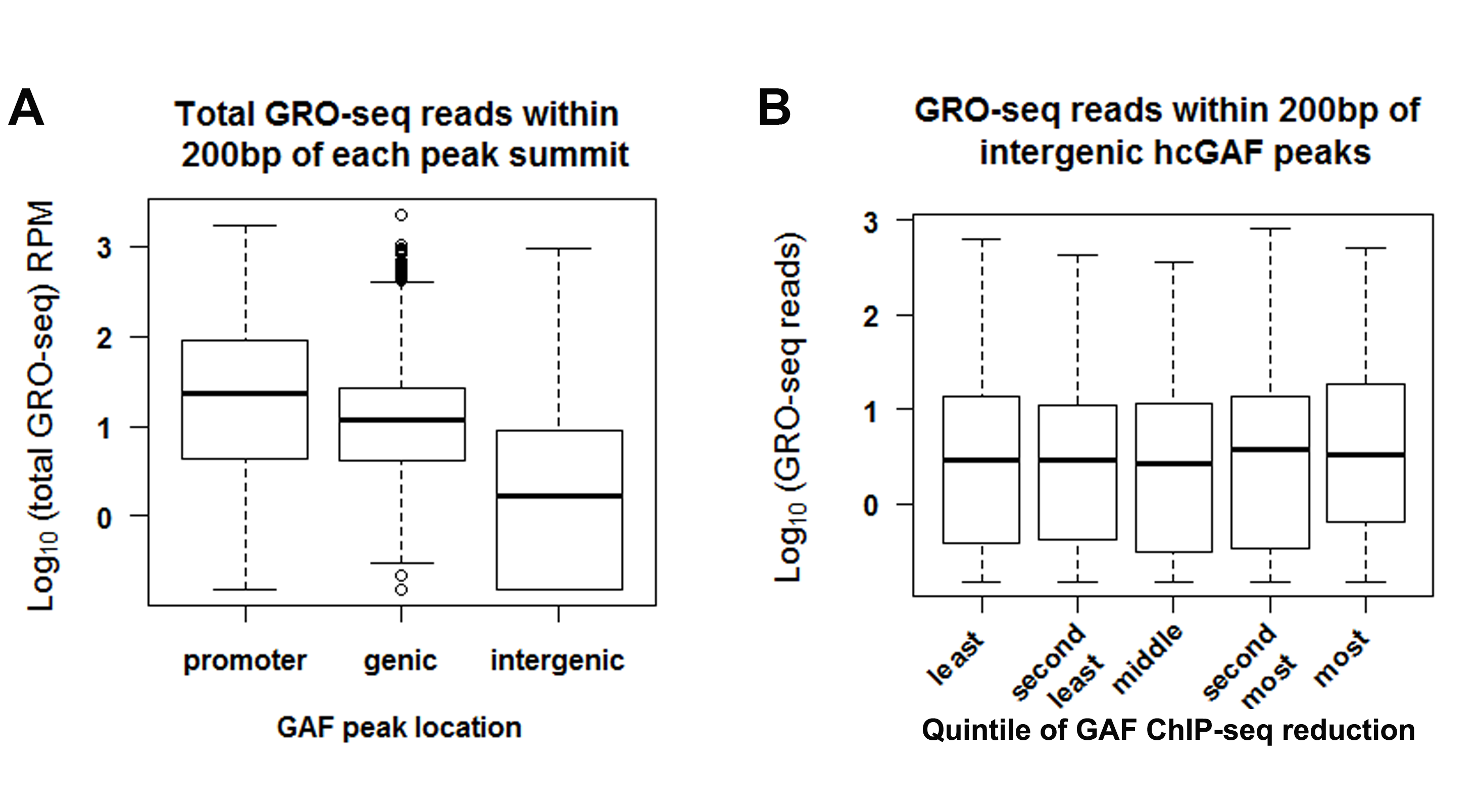

Supplement: S6 Fig — (A) Boxplot showing log10 of the total LacZ-RNAi GRO-seq read within 200bp of promoters, gene body, and intergenic GAF peaks. (B) Boxplot showing log10 of the total LacZ-RNAi GRO-seq read within 200bp of intergenic hcGAF peaks, separated into quintiles based on reduction in GAF binding upon GAF-RNAi. (TIF) [file pgen.1005108.s006.tif]
